# Dr. Osborn’s Carbuncle Treatment

**Published:** 1897-01

**Authors:** E. A. Morris

**Affiliations:** Burton, Texas


For the Texas Medical Journal.

### DR. OSBORN'S CARBUNCLE TREATMENT.

BY E. A. MORRIS, M. D., BURTON, TEXAS.

DR. T. C. OSBORN'S article, in the December number of the JOURNAL, is usefully suggestive in more than one direction. It may be said that it is both negatively and positively so. That is to say, while his experience, as to the practical treatment of the disease in question, is positively useful, his theory as to the etiology of the morbid condition, while evidently ill-founded, is suggestive as to the true theory.

His affirmation that carbuncle "is an evidence of a serious form of protracted indigestion," is not sustained by the character of his remedies, and the results of their use. If the prime cause were indigestion, protracted or otherwise, the drugs used were not therapeutically calculated to remove it. The effects were too rapid, and too complete, to be attributed to the general tonic effect of the prescription. And it was not calculated to relieve, even temporarily, the conditions. As measured by the therapeutic results, the doctor's theory as to causation is erroneous. So measured, however, the deduction is strongly in favor of malarial origin. Because, that part of the treatment on which he lays the greatest stress, is pre-eminently antimalarial. Even the local application, peruvian bark, is that, or nothing. I have known and used, what he calls Kumpe's tonic, for fully as many years as Dr. Osborn, but always and only as an antidote to malaria. And to say that I have used it so long is to say that I have found it efficient as such. If, then, the doctor's success, which certainly is phenomenal, is attributable to the effect of this prescription, the disease is of malarial origin. If he had used no other remedy, the conclusion would be irresistible. But it so happened that he did use another, a chemical which, although he attached very little importance to it, may, nevertheless, be entitled to the entire credit of the cures. I allude to the hyposulphite of soda, which the doctor used as a laxative, but which, as a matter of fact, is, owing to the easily freed sulphurous acid contained, one of the most efficient intestinal antiseptics, and most powerful germicides known to the materia medica.

If the disease is of specific bacterial origin, as I do not doubt, and if the germs find their way into the system from without, through the alimentary canal, we have a *prima facie* case in favor of the soda hyposulphite being the curative agent.

To avail myself of the benefit of the doubt, I shall, in the future, resort to Dr. Osborn's treatment.

In another respect I have to take issue with the doctor. He affirms that the disease is not recurrent.

I have myself suffered three different times with well marked carbuncle. First, after an interval of about twenty-five years, and second, after one of two or three years. So that I am sure one attack does not immunize the system. But this fact does not, in my view, negative the bacterial origin of the disease, nor render necessary any modification of the treatment.

